# Supplementary material for: Comprehensive Annotation and Functional Exploration of MicroRNAs in Lettuce
Source: Front Plant Sci. 2021 Dec 24;12:781836. doi: 10.3389/fpls.2021.781836 (PMC8739914; doi:10.3389/fpls.2021.781836)
Supplement: Supplementary file 2 [file Data_Sheet_8.ZIP › GSM2230749.plot/Lsa-miR408_Lsat_1_v5_gn_1_9501.1_140_TPlot.pdf]

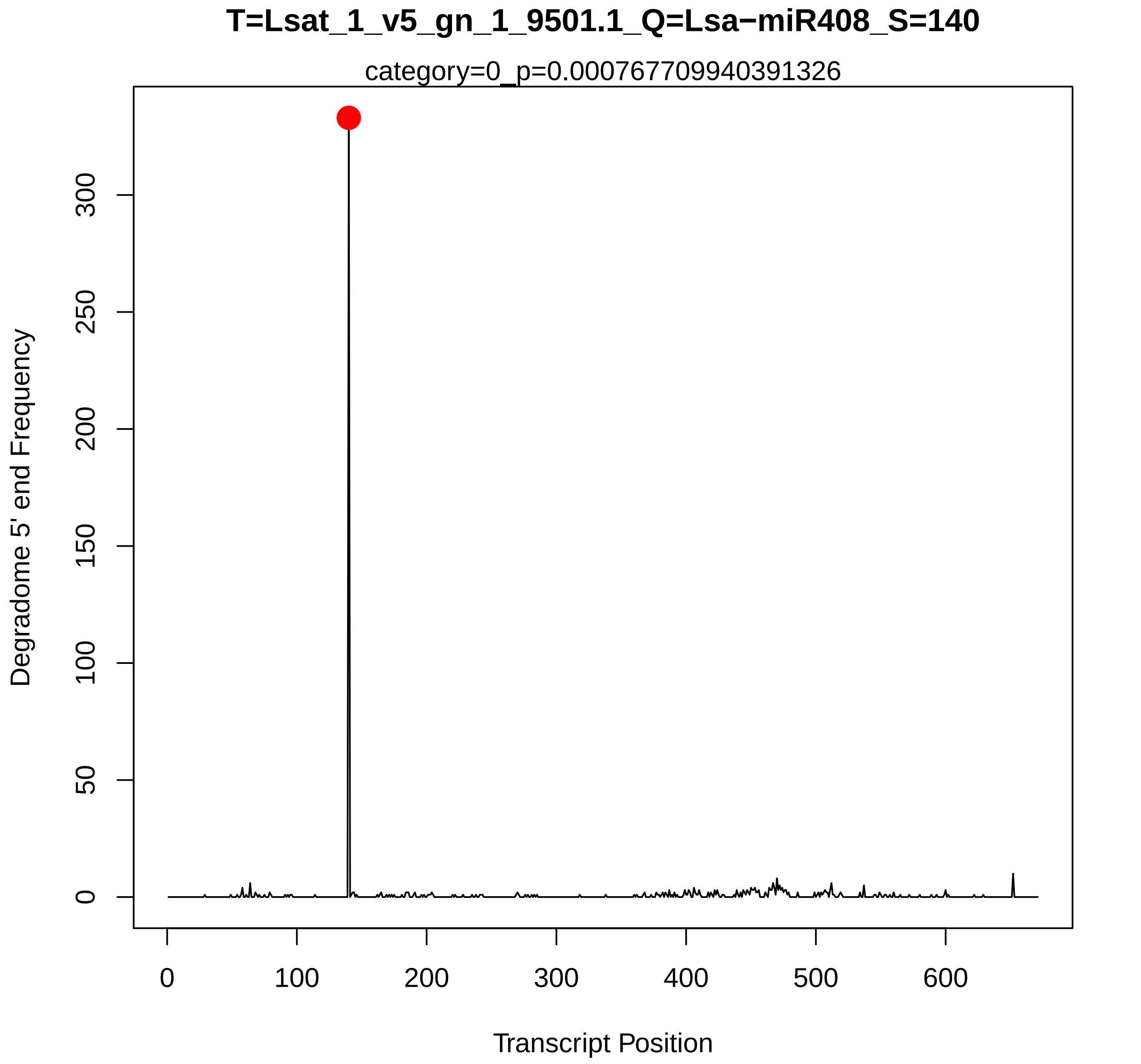

T=Lsat_1_v5_gn_1_9501.1_Q=Lsa−miR408_S=140
category=0_p=0.000767709940391326
Degradome 5' end Frequency
0
50
100
150
200
250
300
0
100
200
300
400
500
600
Transcript Position
